# Analysis of human-oriented facial signals of the domestic dog using eye tracker technology

**DOI:** 10.3389/fvets.2026.1829873

**Published:** 2026-05-13

**Authors:** Julia Sheidin, Sara Youssef Asaad, Samah Ghazawi, Helena Chaloupková, Petra Eretová, Lucie Přibylová, Péter Pongrácz, Anna Zamansky

**Affiliations:** 1 Department of Software Engineering & Information Systems, Braude College of Engineering, Karmiel, Israel; 2 Department of Ethology and Companion Animal Sciences, Faculty of Agrobiology, Food and Natural Resources, Czech University of Life Sciences Prague, Prague, Czechia; 3 Department of Ethology, ELTE Eötvös Loránd University, Budapest, Hungary; 4 Tech4Animals Lab, Computer and Information Sciences Faculty, University of Haifa, Haifa, Israel

**Keywords:** area of interest (AOI), brachycephaly, dog-human communication, dog-human relationship, eye tracker, facial expression, morphology

## Abstract

**Introduction:**

Understanding canine facial signals is crucial for responsible dog ownership and animal welfare, yet how humans visually process these signals in breeds with extreme morphology remains under-researched.

**Methods:**

This study examined visual attention patterns using eye-tracking when humans viewed brachycephalic versus normocephalic dog faces. Forty-four undergraduate students viewed photographs of Boston Terriers (brachycephalic) and Jack Russell Terriers (normocephalic) depicting four emotional contexts: positive (Called by Name, Play) and negative (Separation, Stranger Threat). Eye-tracking measured fixation duration, number of visits, and saccades across eight facial regions (eyes, ears, cheeks, forehead, snout). Participants also self-reported which regions they believed they attended to most.

**Results:**

Brachycephalic dogs elicited 45–46% more visual attention across all frequency- based measures: longer total fixation duration (M = 1103.62 ms, SD = 1364.14 vs. M = 760.08 ms, SD = 1165.99; *p* < 0.001), more fixations, and more visits. Critically, average fixation duration did not differ between breeds, indicating intensive sampling through repeated revisits rather than deeper processing per fixation. Regional analyses using linear mixed-effects models revealed the strongest effects for the ears (Hedges’ g = 0.69–1.59), followed by the eyes, forehead, and snout. The right eye was the only region showing greater attention to normocephalic dogs. Substantial discrepancies emerged between self-reported and objectively measured attention: participants underestimated eye fixations while overestimating snout attention.

**Discussion:**

These findings suggest that extreme brachycephaly is associated with increased visual processing demands, potentially requiring more frequent sampling to interpret facial signals. This may impair signal readability with important implications for animal welfare, veterinary practice, and human-dog communication.

## 1 Introduction

The mutualistic, co-evolutive relationship between domestic dogs *(Canis familiaris)* and humans has been ongoing since the Pleistocene era (70). Within this history, dogs have evolved behavioral strategies that support social interaction with humans, including the use of facial expressions (1). Facial expressions function as visual signals that can convey affective states and intentions (2–4). Humans strongly rely on the facial region when assessing dogs’ internal states, largely irrespective of skull shape (5). This capacity has practical implications: accurate reading of canine signals supports safe and appropriate communication and may strengthen the human-dog bond, whereas misinterpretation of canine signals can increase the risk of negative outcomes, including aggression (6, 7, 72).

Previous studies have shown that humans can infer dogs’ affective states from facial expressions (8–13). What remains less clear is how this interpretation is shaped by breed-typical morphology, especially given the extreme craniofacial variation of modern breeds. Only a small number of studies have addressed morphology-related differences in visual cues and their influence on human observers (5, 14), and existing evaluations of breed-dependent cues have been limited or absent (5, 14). This gap is particularly relevant in brachycephalic dogs, whose extreme craniofacial morphology is characterized by a markedly shortened muzzle, a widened and rounded skull, a prominent forehead, large and protruding eyes, and a flattened facial profile that compresses the spatial arrangement of key features relative to mesocephalic and dolichocephalic breeds (15, 16). These paedomorphic, infant-like proportions have been suggested to elicit nurturing responses and positive affect in human observers (17, 18). However, the same structural changes may constrain the range of facial movements available for social signaling (16, 19, 20) and may also alter how humans perceive their expressions (5, 21, 74). At the same time, while it has been suggested that selective breeding for less extreme signs of brachycephaly would be beneficial for the health of the dogs (22), the extreme phenotypes remain popular. Despite welfare concerns and growing public awareness of health issues associated with brachycephaly (15, 23–25), brachycephalic breeds remain widely popular among dog owners (17), underscoring the importance of investigating how humans visually process their facial signals.

Eye-tracking provides an objective way to quantify visual attention by recording gaze location and movements during stimulus viewing, thereby revealing which regions observers prioritize (26). A common analytical approach involves defining Areas of Interest (AOIs)—specific regions within the visual stimulus—and measuring how much time and how many fixations observers allocate to each, thereby identifying which elements attract the most attention and are considered most informative for interpreting the presented information (26). In human emotion research, gaze allocation to diagnostic facial regions—often the eye region—has been linked to accurate emotion classification (27–33). However, self-reports of attending to the eyes can overestimate actual eye-directed gaze, as eye-tracking shows that viewers often inspect the whole face rather than maintaining direct eye contact (34). Eye-tracking has also been widely adopted in interspecies research, including studies of dogs’ perception of human visual signals (35–41).

By contrast, relatively few studies have examined how humans visually scan dog faces when judging canine emotions or behavior. Guo et al. (42) found that individuals without pets predominantly fixate on dogs’ eyes (followed by the nose), with little attention to the mouth, and that fixations on dog mouths are fewer than on human or monkey mouths. Correia-Caeiro et al. (43) further suggested that a human-centric focus on the eyes and mouth may overlook canine-specific cues (e.g., ear position), potentially impairing emotion assessment. Age- and valence-related differences have also been reported, with children spending more time viewing faces depicting negative than positive affective states (44). Critically, it remains unknown whether gaze strategies and the facial regions supporting judgments differ systematically when observers view dogs with extreme craniofacial morphologies.

To address this gap, in a new study we incorporated eye-tracking technique with stimulus material previously collected by Eretová et al. (5). That study examined recognition of contextual signals in brachycephalic Boston Terriers and normocephalic Jack Russell Terriers across positive and negative situations, but questionnaire-based self-reports of areas of interest (AOIs) did not show clear breed differences (5). Here, we selected eight images (four per breed type) to replicate the self-report component and extend it with objective gaze measurement, which provides a continuous record of attention (fixation duration, scan sequence, and returns to regions) and is less susceptible to memory- and expectation-driven biases in retrospective reporting. Consistent with human face research showing discrepancies between perceived and measured gaze allocation and effects of feature clarity/eye visibility on within-face gaze distribution (34, 73), we test whether such dissociations also occur for canine stimuli—particularly for dogs with markedly altered facial structure.

We recorded eye movements with a new sample of participants to test whether craniofacial morphology is associated with (i) overall attention to the face, (ii) prioritization of specific facial regions, and (iii) different self-reported prioritization of AOI in comparison with eye-tracking measurements.

We further make the following hypotheses:

- Processing Intensity: Participants will exhibit increased attention (i.e., longer fixation duration, higher frequency of visits, more saccades, time to first fixation, and viewing order between AOIs) toward brachycephalic dogs, potentially associated with greater demands on visual processing of their facial signals.
- Feature Prioritization: The eye region will be a focal point of attention across breeds more than other AOIs, but with higher intensity (longer duration and more frequently) in brachycephalic dogs compared to normocephalic dogs.
- Self-reported prioritization of specific facial regions will align with prioritization of facial regions recorded via eye-tracking.

## 2 Materials and methods

### 2.1 Eye-tracking technology

Eye-tracking is a technology that measures and records where a person is looking, how long they look at specific areas, and how their gaze moves across a visual scene (45). It works by projecting near-infrared light onto the eye; cameras then detect the reflections from the cornea and pupil, and algorithms use these reflections to calculate the precise point of gaze in real time. The most commonly used method is corneal reflection tracking. A light source illuminates the eye, creating a reflection (the “glint”) on the cornea. The relative position of this glint to the center of the pupil is used to determine gaze direction (46). Modern systems can track both eyes simultaneously at sampling rates of up to 1,200 Hz, allowing very precise temporal resolution (47).

Several key metrics are derived from eye-tracking data. Fixations refer to periods during which the gaze is held relatively still on a specific location, indicating active visual attention and cognitive processing. Saccades are the rapid eye movements that occur between fixations, as the gaze shifts from one point of interest to another. Areas of Interest (AOIs) are predefined regions within a visual scene that allow researchers to quantify how much attention is directed toward particular elements. Time to first visit reflects how quickly the gaze reaches a given AOI, serving as an indicator of the salience or attentional priority of that region. Together, these metrics provide a detailed, objective account of visual attention that cannot be obtained through self-report measures alone (48).

### 2.2 Participants

Forty-five undergraduate students from Braude College of Engineering participated in the study. The convenience sample of technology students was chosen to represent non-expert observers. Sample size was determined based on previous eye-tracking studies examining human visual attention to animal faces, which have reported robust effects with comparable or smaller samples (39, 42, 43). These students, all studying technological subjects, volunteered for the experiment after advertisements were posted throughout the college. Among them were 17 females and 28 males, with an average age of 22.61 years (SD = 2.09). Dog owners accounted for 47.73% of all participants, and 13.64% of respondents reported previously owning at least one brachycephalic dog. The study was approved, as required, by the Institutional Research Ethics Committee at Braude College of Engineering (approval number 2042–05). The participants provided informed consent to participate in this study.

### 2.3 Stimuli

**Figure 1:**
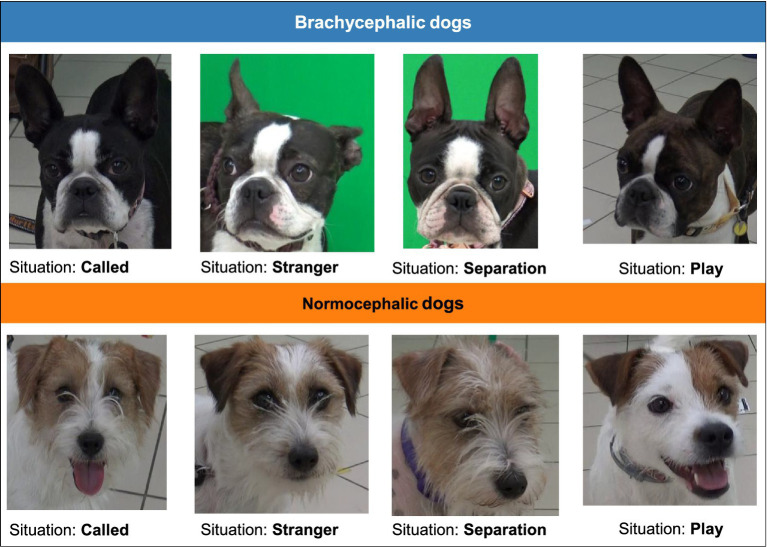
The photos used in the experiment (4 brachycephalic and 4 normocephalic dogs).

Our dataset consisted of 8 images (presented in Figure 1) drawn from the experiment conducted by Eretová et al. (5), which documented the behaviors of two dog breeds in artificial setting of the ethological laboratory at Department of Ethology, ELTE: Boston Terriers (*n* = 16), representing brachycephalic dogs, and Jack Russell Terriers (*n* = 7), representing normocephalic dogs. Breed was the primary inclusion criterion, selected to represent the two extremes of the cephalic spectrum; additionally, the dogs had to be over 1 year of age. Exclusion criteria were set as dogs showing any signs of ongoing health problems that would negatively affect their natural responses to the experimenters (such as significant breathing difficulties, ongoing illness, or injury) or exacerbated distress response to the experimental design. From a larger set of video recordings collected in that study, eight still frames were selected—one per breed per situation—based on image clarity, frontal head orientation, and representativeness of each dog’s behavioral display in the given context [for full details of the original recording protocol, see (5)]. Each image was edited and cropped to focus exclusively on the dog’s face, removing the body and background context. No further selection criteria (e.g., age, sex, or body size of the dogs) were applied beyond breed classification, and individual variation in features such as coat markings, facial wrinkles, or slight head tilt could not be fully controlled across the eight stimuli. Images of the dog’s face depicted one of four situations.

Dogs have been recorded in 4 situations, for full details see Ref. (5).

Situation 1 – Dog called by name (“Called”): Experimenter 1 called the dog by its name while Experimenter 2 recorded the dog’s responses with a handheld camera.

Situation 2 – Play (“Play”): The dog was instructed to play with a tennis ball while on a leash. Experimenter 1 presented the ball without giving it to the dog. Responses were recorded using both tripod and handheld cameras, ensuring that both the ball and Experimenter 1 remained hidden. Prior to recording this situation, dogs were familiarized with the tennis ball and enticed to freely play with it to ensure a positive response before being leashed again for the recording [for more detail, please see the full experiment breakdown in Ref. (5)].

Situation 3 – Separation (“Separation”): The owners left a dog in an experimental room for a brief examination, while Experimenter 1 was present and kept the dog on leash, maintaining a distance of at least 2 meters.

Situation 4 – Threatened by a stranger (“Stranger”): Experimenter 2 threatened the dog by crouching, taking wide steps, and maintaining eye contact. Experimenter 1 captured the image while keeping Experimenter 2 out of the frame, and Experimenter 2 remained silent. After the brief ‘threatening’ approach, Experimenter 2 initiated play session with the dog, to dissolve any remaining stress.

The stimuli were obtained in accordance with the guidelines for the use of animals in research, as outlined by the Association for the Study of Animal Behavior (ASAB). The situations were chosen as everyday social contexts in a dog’s life. The procedure was fully non-invasive. The methodology was reviewed and approved by the Animal Welfare Committee of Eötvös Loránd University (Certificate number PEI/001/1056–4/2015). Informed consent was obtained from every dog owner who participated in this part of the experiment.

### 2.4 Procedure

After a welcome and a brief introduction to the study’s purpose, participants were informed that they would take part in an experiment investigating how people understand the visual signals of extremely brachycephalic (short-nosed) dogs by evaluating two breeds using eye-tracking technology. They were instructed to observe sets of photographs featuring dog faces and answer follow-up questions (these same instructions would later appear on the computer screen). Participants were informed that their eye movements would be monitored and that their responses would remain anonymous. They were also informed that participation was voluntary and asked to sign an informed consent form. Next, participants completed demographic questions regarding their gender and age. They could provide their name and email address if they wished to receive feedback on their answers and communicate with the research team, though this was not mandatory. All personal information provided by participants was anonymized and stored separately for later examination.

Participants then proceeded with the experimental procedure, where they were presented with eight images of dog faces, both brachycephalic and normocephalic (four per type). To maintain methodological consistency with Eretová et al. (5), images were presented in a fixed order that alternated between breeds and situations: Brachycephalic in the Called situation, Brachycephalic in the Stranger situation, Normocephalic in the Called situation, Brachycephalic in the Play situation, Normocephalic in the Separation situation, Normocephalic in the Stranger situation, Brachycephalic in the Separation situation, and Normocephalic in the Play situation. Participants only looked at the still photos shown in Figure 1. While this fixed order ensured replication consistency, we acknowledge that counterbalancing was not implemented and recommend this methodological enhancement for future studies to control for potential order and fatigue effects. For each image, participants first viewed the dog face while their eye movements were recorded, then transitioned via mouse click to answer three questions administered in a Google Form. As in Eretová et al. (5), they identified the situation they believed the dog was in, such as being called by its name, playing, being separated from its owners, or being threatened by a stranger. Additionally, they indicated which areas of the dog’s face they focused on (eyes, ears, cheeks, forehead, and snout area) to discern the dog’s emotions and current state, selecting all responses via the Google Forms interface. This sequential design enabled the collection of both objective gaze data and subjective self-reports while maintaining consistency with the replicated study. After submitting an answer and proceeding to the next question, participants could not return to the previous one. To transition to the next image, participants pressed “F10,” and they moved from each image to its associated questionnaire by mouse click. Each session lasted for 15 to 20 min.

### 2.5 Eye tracking apparatus and measures

#### 2.5.1 Apparatus

The experiments were conducted in a quiet room, with one participant at a time. Each participant was seated in front of a 24-inch screen displaying full HD (1920×1080) resolution, positioned 65 cm from their eyes. Eye movements were recorded using a Tobii Pro Spark eye tracker (Tobii AB, founded 2001 Stockholm, Sweden; set at sampling rate: 60 Hz) mounted at the bottom of the display monitor. A webcam positioned above the screen captured participants’ facial expressions and behavior to facilitate monitoring, synchronization, and quality assurance. The experimental protocol was run using Tobii Pro Lab (version 1.232). At the beginning of each session, the eye-tracking system was calibrated for each participant using a 9-point calibration protocol, ensuring precision and accuracy within 0.5 degrees of visual angle. Throughout the entire session, eye tracking was performed to identify the specific areas of the dog’s face that participants focused on, including the duration and frequency of their gaze. No equipment was attached to the participants.

#### 2.5.2 Areas of interest

Areas of Interest (AOIs) are specific regions within an image or visual stimulus where researchers measure participants’ attention. In eye-tracking studies, AOIs help determine how much time or attention participants spend on different parts of an image (26). By analyzing these regions, we can gain insights into which elements attract the most attention and are considered most important for interpreting the information presented.

**Figure 2:**
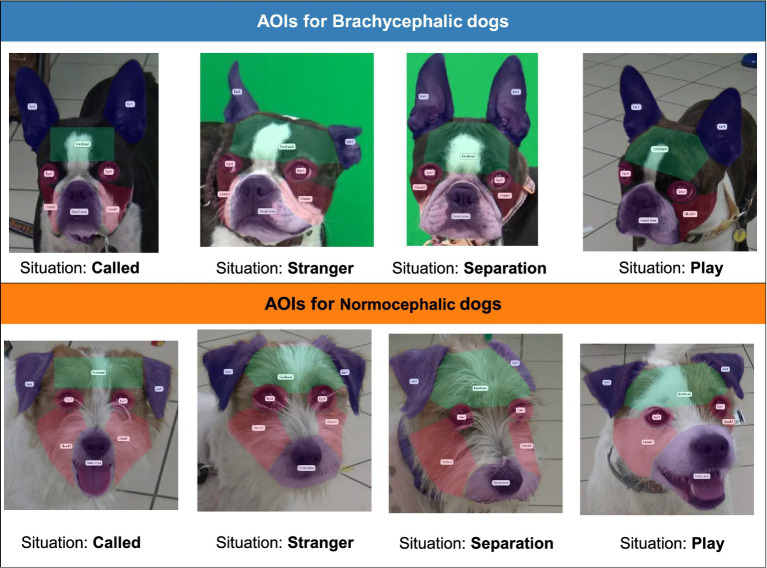
AOIs delineations for Brachycephalic and Normocephalic dogs across the four situations.

Eight AOIs were manually delineated for each image within the Tobii Pro Lab eye-tracking analysis software, using polygonal shapes to optimize spatial accuracy. These AOIs corresponded to key facial regions: the left and right eyes (eye1 = dog’s left eye, eye2 = dog’s right eye), left and right ears (ear1 = dog’s left ear, ear2 = dog’s right ear), left and right cheeks (cheek1 = dog’s left cheek, cheek2 = dog’s right cheek), forehead, and snout area. To reduce potential gaze-duration bias associated with AOI dimensions, all AOIs were defined to be roughly similar in size. Since the absolute sizes of certain facial regions varied across the different dogs and images, AOI boundaries were adjusted individually for each image to ensure full coverage of the target region. Our analytical approach focused on relative comparisons within breed types and situations, and employed multiple metrics (fixation duration, number of visits, saccade count) to provide converging evidence less sensitive to size-related artifacts. See Figure 2 for AOI overlays demonstrating the delineation approach for each image. It should be noted that the camera angle in the brachycephalic stimuli caused a slight tilt in the dog’s face, making the left eye less visible than the right, which may have influenced fixation patterns in this region.

#### 2.5.3 Metrics

An eye tracker captures gaze information through three main components: fixations, visits, and saccades (26). Fixations occur when a person focuses their gaze on a specific location on the screen during a visit to an AOI. Saccades, on the other hand, refer to the rapid movements of the gaze from one fixation point to another. The number of visits indicates the separate occasions when a participant’s gaze enters an AOI during the session; each visit may include one or more fixations. This distinction helps differentiate between sustained attention and repeated checking. Since we are analyzing engagement with specific elements, such as the dog’s facial features, we primarily focus on comprehensive fixation data for greater validity. Fixation data is considered the most common and direct measure of attention. By examining this data, we can identify patterns in viewer attention and explore the order in which participants view different AOIs. The metrics used in this study are detailed in Table 1.

**Table 1:** Description of basic eye tracking measures used in the study.

Measure	Description
Fixation Duration	Reflects how long a participant’s gaze remains focused on a specific AOI—the most common and direct measure of attention.
Average Fixation Duration	The mean duration of fixations within an AOI indicates how deeply each look is processed.
Total Fixation Duration	The sum of all fixation durations to a specific AOI.
Number of Fixations	The number of fixations occurring in an AOI tells you how often a region draws attention, regardless of how long each fixation lasts.
Time to First Fixation	The time elapsed from the start of the trial until the participant first looks at the AOI.
Number of Visits	The number of distinct times a participant’s gaze enters an AOI during the session, regardless of the number of fixations per entry, helps distinguish sustained attention from repeated checking.
Number of Saccades	The number of rapid eye movements between fixations within the region.
Viewing Order	The temporal sequence in which each AOI received its first fixation, ranked from 1 (first fixated AOI) to 8 (last fixated AOI).

The fixation feature was calculated using the attention filter in Tobii Pro Lab (Ver. 1.241). We used the Velocity-Threshold Identification (I-VT) fixation classification algorithm, a velocity-based method (75) that categorizes fixations and saccades based on velocity. If the velocity exceeds 1,000/s, it is classified as a saccade; otherwise, it is classified as a fixation.

#### 2.5.4 Data processing

Prior to conducting the data analysis, we implemented a data processing procedure. First, we eliminated all gaze samples where the eye was not detected, which occurred due to tracking errors or blinks. We also excluded records from visits to different AOIs with viewing times less than 80 milliseconds, which approximates the minimum time needed to consciously process visual information from directly fixated stimuli (49). This elimination resulted in the removal of nearly a third of the collected data. Additionally, data from participant #10 were excluded from our analysis due to her age, which was significantly older than the other participants’ (resulting in *N* = 44).

#### 2.5.5 Statistical analysis

All statistical analyses were conducted in Python 3.12 using Jupyter Notebook. Data manipulation and descriptive statistics were performed using pandas (version 1.5.3) and NumPy (version 1.24.3). Visualization was created using Matplotlib (version 3.7.1).

General Results. Descriptive statistics were calculated for all variables, including frequencies and percentages for categorical variables (gender, dog ownership status) and means with standard deviations for continuous variables (eye-tracking metrics). Overall attention differences (aggregated across all AOIs) were calculated from descriptive statistics and are reported as means, standard deviations, and percentage differences.

Eye-Tracking Metrics. Since each participant viewed all image types, the data followed a mixed factorial repeated-measures design. To account for repeated measures across participants and experimental conditions, the Linear Mixed-Effects Models (LMMs) were fitted separately for each of the eight AOIs using Python’s statsmodels package (version 0.14.0) (50). Each model included breed (brachycephalic vs. normocephalic) as the fixed effect and random intercepts for participants to account for repeated measures. The dependent variables are: (1) total fixation duration (ms), (2) average duration of fixations (ms), (3) number of fixations, (4) number of visits, (5) time to first visit, and (6) the number of saccades within each AOI. Degrees of freedom were estimated based on the number of independent participant units (*N* = 44, df = 42 for fixed effects within each regional model). *p*-values were corrected for multiple comparisons across the eight AOIs using the False Discovery Rate (FDR) method (51). Effect sizes were calculated using Hedges’ g to provide standardized measures of the magnitude of breed differences.

For all linear mixed-effects models, we report the unstandardized regression coefficient (*β*) representing the difference between breeds, standard error (SE), t-value, degrees of freedom (df = 42 based on *N* = 44 participants), FDR-corrected *p*-values (p_FDR), and Hedges’ g as a standardized effect size. Positive values indicate higher values for brachycephalic dogs; negative values indicate higher values for normocephalic dogs.

Viewing Order. A separate LLM was conducted to analyze the viewing order of the AOIs. Viewing-order ranks (ordinal) were analyzed with Friedman tests, followed by Wilcoxon signed-rank tests with Holm correction to identify AOI differences and breed effects on initial fixation sequence.

Self-Reported vs. Measured Attention. To compare participants’ perceived focus on facial features with their actual gaze behavior, we analyzed both self-reported AOI selections (eyes, ears, cheeks, forehead, and snout area) and objective eye-tracking data from Tobii Pro. Participants indicated which AOI they believed they focused on most, while the Tobii system recorded total fixation duration within each AOI. Eye-tracking engagement was quantified as the number of whole fixations per AOI, and fixation proportions were computed by dividing AOI-specific fixations by the total fixations in each trial. Self-reported AOI proportions were then compared with Tobii-derived proportions using 95% bootstrap confidence intervals (B = 2000). Discrepancies between self-reported and Tobii AOI categories were assessed using the Stuart-Maxwell and Bowker tests for paired multicategory responses. Exposure-normalized fixation rates (fixations per second) were calculated using maximum trial viewing time. Where sufficient data were available, fixation proportions were further summarized by breed, gender, and ownership, and mixed-effects models with random intercepts for trials were used to estimate AOI-level effects.

All results were considered statistically significant at *p* < 0.05. Data visualization was performed using the Matplotlib and Seaborn packages in Python.

## 3 Results

### 3.1 General results

#### 3.1.1 Participant characteristics and descriptive statistics

A total of 44 unique participants: male (*n* = 28, 63.6%) and female (*n* = 16, 36.4%) participated in this study. Regarding dog ownership, 44 participants provided status: no dog (*n* = 23, 52.3%), Normocephalic owner (*n* = 15, 34.1%), and Brachycephalic owner (*n* = 6, 13.6%). Exploratory analyses revealed no significant associations between participant characteristics (age, gender, dog ownership status) and eye-tracking measures, suggesting that the observed attention patterns were not moderated by individual differences.

Participants exhibited significant variability in eye-tracking measures across all images. The mean total fixation duration was 929.12 milliseconds (SD = 430.14), with an average fixation duration of 322.73 milliseconds (SD = 97.61). On average, there were 2.88 fixations per image (SD = 1.14). Additionally, the mean total visit duration was 1,038.22 milliseconds (SD = 456.92), with an average visit duration of 411.55 milliseconds (SD = 147.04).

#### 3.1.2 Overall attention differences across all AOIs

**Figure 3:**
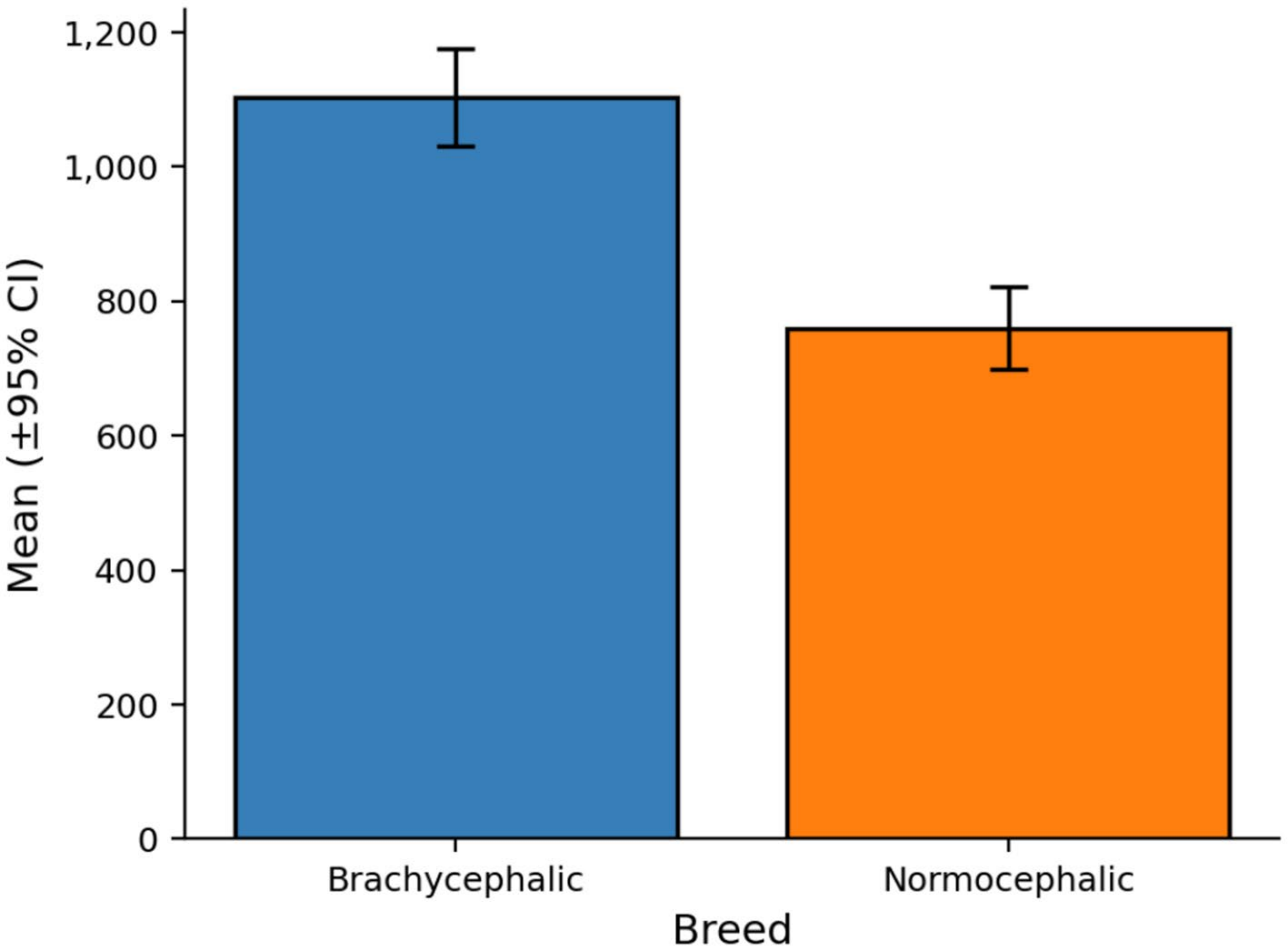
Mean total fixation duration by breed, using brachycephalic (blue) vs. normocephalic (orange) color scheme. Each bar shows the group means with a 95% CI.

Participants allocated significantly more visual attention to brachycephalic dogs compared to normocephalic dogs across multiple eye-tracking metrics (aggregated across all facial regions). Total fixation duration was approximately 45% longer for brachycephalic dogs (*M* = 1103.62 ms, *SD* = 1364.14, *n* = 1,364) than normocephalic dogs (*M* = 760.08 ms, *SD* = 1165.99, *n* = 1,408) as shown in Figure 3. Similarly, participants made approximately 46% more fixations on brachycephalic dogs (*M* = 3.43, *SD* = 3.89) compared to normocephalic dogs (*M* = 2.35, *SD* = 3.29), and approximately 42% more visits to images of brachycephalic dogs (*M* = 2.87, *SD* = 3.02) compared to normocephalic dogs (*M* = 2.03, *SD* = 2.62). The number of saccades within AOIs was also approximately 46% higher for brachycephalic dogs (*M* = 0.72, *SD* = 1.57) compared to normocephalic dogs (*M* = 0.49, *SD* = 1.25). In contrast, average fixation duration did not differ substantially between breeds (brachycephalic: *M* = 326.28 ms, *SD* = 179.55; normocephalic: *M* = 322.19 ms, *SD* = 212.94), representing only a 1.3% difference. Time to first visit showed a modest 12% increase for brachycephalic dogs (*M* = 2979.23 ms, *SD* = 3614.40) compared to normocephalic dogs (*M* = 2670.07 ms, *SD* = 3915.94). The minimal difference in average fixation duration suggests that the increased attention to brachycephalic dogs reflected more frequent sampling rather than deeper processing per individual fixation.

No statistically significant effects emerged for either gender or dog ownership. Thus, within this dataset, neither variable appears to account for additional variance in the eye-tracking measures once breed is included in the model.

### 3.2 Eye tracking metrics

To analyze differences in visual attention across AOIs by breed, we conducted linear mixed-effects models for each area.

#### 3.2.1 Fixations

**Figure 4:**
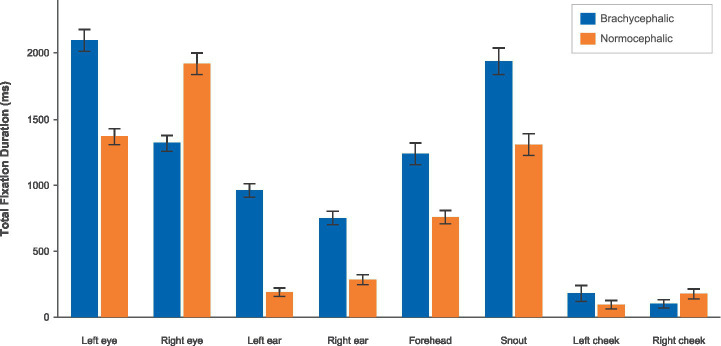
Total fixation duration by breed and AOI using the brachycephalic (blue) vs. the normocephalic (orange) color scheme.

Participants fixated significantly more on most facial regions of brachycephalic dogs than on normocephalic dogs (see Figure 4; Table 2).

**Table 2:** Linear mixed-effects model results comparing total fixation duration (milliseconds) across AOI between brachycephalic and normocephalic dogs.

Facial region	*β*	*SE*	*t*	*df*	*p*FDR	*Hedges’ g*
Left ear	760.06	64.60	11.77	42	<0.001	1.12
Right ear	451.40	71.54	6.31	42	<0.001	0.59
Left eye	740.36	135.56	5.46	42	<0.001	0.51
Forehead	489.78	105.88	4.63	42	<0.001	0.45
Right eye	−603.97	125.85	−4.80	42	<0.001	−0.42
Snout	628.60	128.68	4.88	42	<0.001	0.39
Left cheek	101.91	36.04	2.83	42	0.005	0.24
Right cheek	−72.13	31.11	−2.32	42	0.020	−0.18

**Figure 5:**
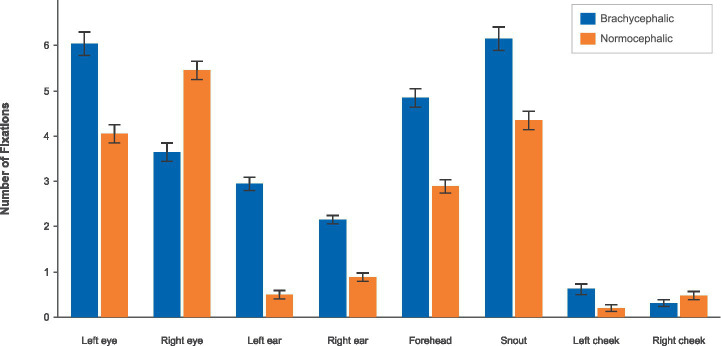
Number of fixations by breed and AOI using the brachycephalic (blue) vs. the normocephalic (orange) color scheme.

The pattern of results for the number of fixations closely mirrored that of total fixation duration (see Figure 5; Table 3). Seven out of eight facial regions showed significant breed differences after FDR correction.

**Table 3:** Linear mixed-effects model results comparing the number of fixations across AOI between brachycephalic and normocephalic dogs.

Facial region	*β*	*SE*	*t*	*df*	*p*FDR	*Hedges’ g*
Left ear	2.39	0.18	13.50	42	<0.001	1.34
Right ear	1.31	0.17	7.83	42	<0.001	0.71
Forehead	1.93	0.35	5.57	42	<0.001	0.56
Left eye	2.03	0.35	5.79	42	<0.001	0.53
Right eye	−1.80	0.32	−5.62	42	<0.001	−0.49
Left cheek	0.41	0.09	4.47	42	<0.001	0.45
Snout	1.76	0.39	4.55	42	<0.001	0.37
Right cheek	−0.17	0.09	−1.93	42	0.053	−0.19

In stark contrast to the frequency-based measures, average fixation duration showed minimal regional differences between breeds. Mixed-effects models revealed only one significant difference after FDR correction: the snout area showed slightly longer average fixations for brachycephalic dogs (*β* = 29.01 ms, *SE* = 10.39, *t*(42) = 2.79, *p*FDR = 0.042, Hedges’ *g* = 0.25). All other regions showed non-significant differences (all *p*FDR > 0.18), with effect sizes ranging from *g* = −0.20 to *g* = 0.12. The forehead showed virtually no difference (*β* = −0.29 ms, *SE* = 10.40, *t*(42) = −0.03, *p*FDR = 0.978, *g* = −0.00).

The pattern of null findings for average fixation duration, alongside significant effects on fixation frequency, suggests that participants viewed brachycephalic dog faces more often but did not spend more time per fixation. This indicates increased cognitive effort through multiple revisits rather than deeper processing.

#### 3.2.2 Visits

**Figure 6:**
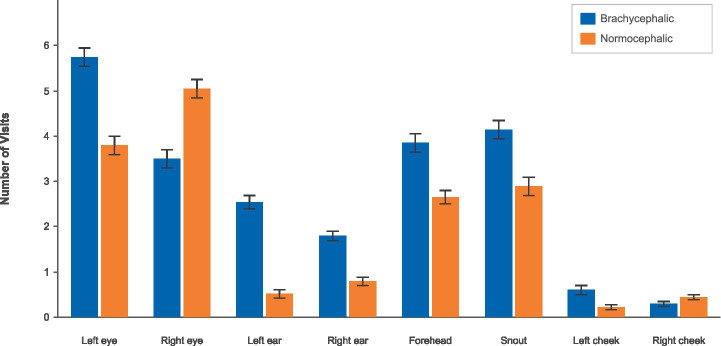
Number of visits by breed and AOI using the brachycephalic (blue) vs. the normocephalic (orange) color scheme.

Results for the number of visits demonstrated the same pattern with even stronger effect sizes (see Figure 6; Table 4).

**Table 4:** Linear mixed-effects model results comparing the number of visits across AOI between brachycephalic and normocephalic dogs.

Facial region	*β*	*SE*	*t*	*df*	*p*FDR	*Hedges’ g*
Left ear	2.02	0.12	16.26	42	<0.001	1.59
Right ear	1.02	0.13	7.86	42	<0.001	0.70
Left eye	1.90	0.32	6.01	42	<0.001	0.55
Forehead	1.19	0.23	5.28	42	<0.001	0.52
Snout	1.27	0.23	5.49	42	<0.001	0.47
Right eye	−1.50	0.28	−5.42	42	<0.001	−0.47
Left cheek	0.36	0.08	4.30	42	<0.001	0.43
Right cheek	−0.16	0.08	−1.98	42	0.048	−0.20

**Figure 7:**
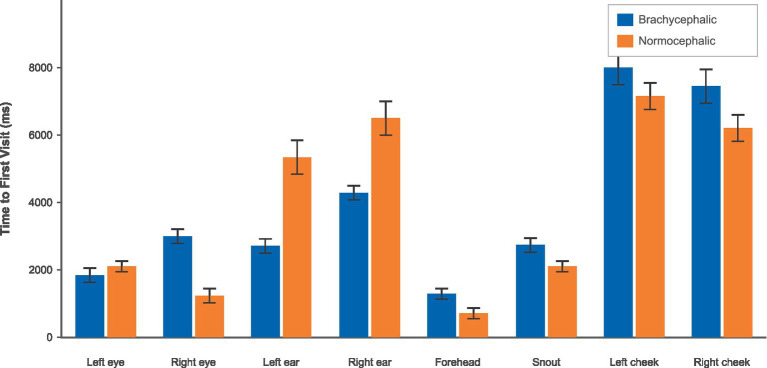
Time to first visits by breed and AOI using the brachycephalic (blue) vs. the normocephalic (orange) color scheme.

Time to first visit showed a more complex regional pattern (see Figure 7). Four out of eight regions showed significant breed differences after FDR correction. The forehead showed a trend toward faster attention for brachycephalic dogs (pFDR = 0.100), while the left eye and both cheeks showed no significant differences in time to first visit (all pFDR > 0.29). These patterns suggest that the ears of brachycephalic dogs, which showed the strongest overall attention effects, also captured initial attention more rapidly.

It is important to note that the first AOI visited was the forehead for both breeds. For the normocephalic breed, the order of first visits to the AOIs was as follows: forehead, right eye, left eye, snout area, left ear, right cheek, right ear, and left cheek. In contrast, for the brachycephalic breed, the order of first visits to the AOIs was: forehead, left eye, left ear, snout area, right eye, right ear, right cheek, and left cheek.

#### 3.2.3 Saccades

**Figure 8:**
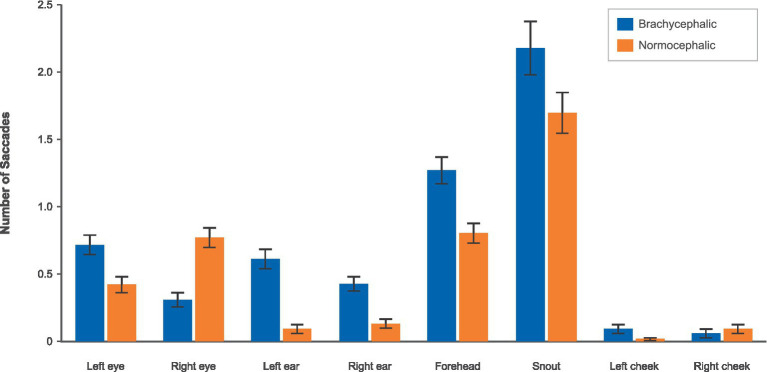
Number of saccades by breed and AOI using the brachycephalic (blue) vs. the normocephalic (orange) color scheme.

Analysis of saccadic eye movements within AOIs revealed a pattern consistent with other frequency-based measures (see Figure 8; Table 5). Seven out of eight facial regions showed significant breed differences after FDR correction.

**Table 5:** Linear mixed-effects model results comparing the number of saccades across AOI between brachycephalic and normocephalic dogs.

Facial region	*β*	*SE*	*t*	*df*	*p*FDR	*Hedges’ g*
Left ear	0.53	0.08	6.68	42	<0.001	0.69
Right eye	−0.46	0.09	−5.19	42	<0.001	−0.52
Right ear	0.30	0.07	4.54	42	<0.001	0.45
Left eye	0.30	0.09	3.15	42	0.003	0.31
Left cheek	0.08	0.03	2.81	42	0.007	0.30
Forehead	0.47	0.17	2.85	42	0.007	0.29
Snout	0.48	0.23	2.06	42	0.045	0.18
Right cheek	−0.03	0.03	−1.01	42	0.311	−0.11

In summary, frequency-based measures (total fixation duration, number of fixations, number of visits, number of saccades) showed consistent breed differences across seven out of eight AOIs, with brachycephalic dogs eliciting substantially more attention. In contrast, average fixation duration showed minimal breed differences, and time to first visit revealed that brachycephalic ears captured attention more rapidly.

### 3.3 Viewing order

Beyond individual AOI attention, we analyzed the temporal sequence of visual scanning.

Participants’ fixation sequences differed significantly across areas of interest (AOIs). A Friedman test on balanced trials (Participant × Image as blocks) showed a strong overall effect of AOI on viewing-order rank, χ^2^(3, *N* = 279) = 205.84, *p* < 0.001, indicating that certain AOIs tended to be fixated earlier than others.

Follow-up Wilcoxon signed-rank tests (paired within trial), corrected using Holm’s method, confirmed specific ordering differences. The Forehead was fixated earlier than the snout area (V = 5,953, pHolm < 0.001), earlier than the left eye (V = 8,238, pHolm < 0.001), and earlier than the right eye (V = 8,599, pHolm < 0.001). Right eye (V = 14018.5, pHolm < 0.001) and left eye (V = 14,744, pHolm < 0.001) were also fixated earlier than the snout area. These pairwise results align with the overall finding and indicate a consistent prioritization of facial AOIs over the snout area during early fixations.

To examine breed effects on the initial fixation sequence, we conducted breed-stratified Friedman tests using the same balanced-within-breed procedure. In brachycephalic dogs, AOIs differed in viewing order, χ^2^(3, *N* = 145) = 77.81, p < 0.001, with Holm-corrected Wilcoxon tests indicating Forehead was fixated earlier than the snout area (V = 1764.0, pHolm < 0.001) and earlier than the left ear (V = 2461.5, pHolm < 0.001). The left eye was fixated earlier than the snout area (V = 3102.0, pHolm < 0.001) and earlier than the forehead (V = 3135.5, pHolm < 0.001). The normocephalic subset did not retain enough balanced trials to support a stable within-breed test; therefore, no reliable within-breed pairwise comparisons are reported. Exploratory between-breed Mann–Whitney comparisons likewise did not meet the minimum balanced-data requirements for inclusion.

**Figure 9:**
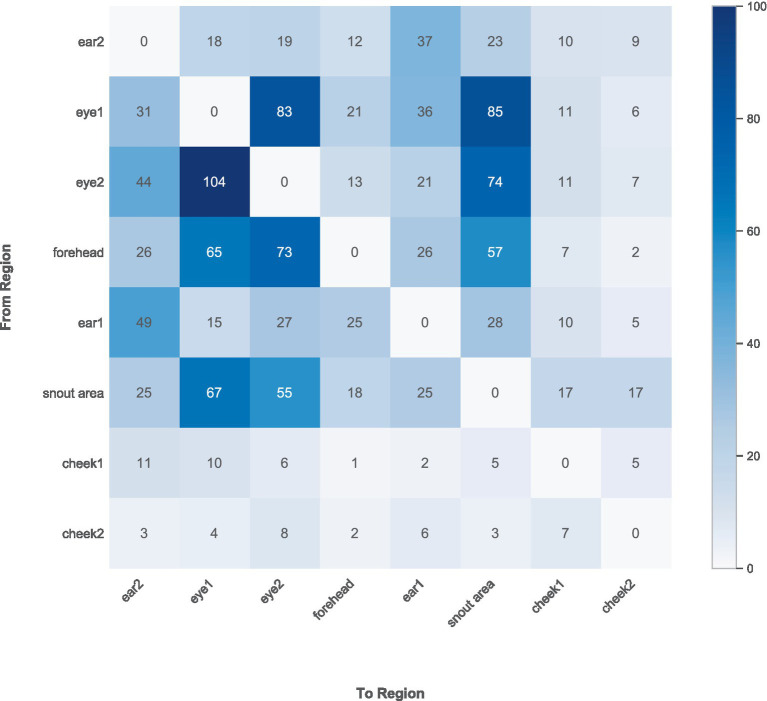
Transition frequency matrix between AOIs combined for both breeds.

To better understand how participants visually explored the dog faces, we examined both the frequency of transitions between AOIs and the typical viewing sequence across AOIs, combined for both breeds. The rate at which participants’ visual attention moved from one AOI to another was calculated and is shown in the frequency matrix below (see Figure 9), where each cell indicates how often participants moved their attention from one AOI (row) to another AOI (column). Тhe most frequent transitions occurred between the eyes (left eye and right eye), as well as between the eyes and the snout area or forehead. This suggests that participants often shifted their visual attention among these central facial features.

### 3.4 Comparison between self-reported and objectively measured attention

#### 3.4.1 Self-report vs. Tobii number of visits (combined across breeds)

We compared attention allocation across five AOIs: eyes, ears, cheeks, forehead, and snout area using two measures aggregated across all participants and images: self-reported AOIs and Tobii number-of-visits proportions (each AOI’s share of total visits within participant-by-image, then averaged).

**Figure 10:**
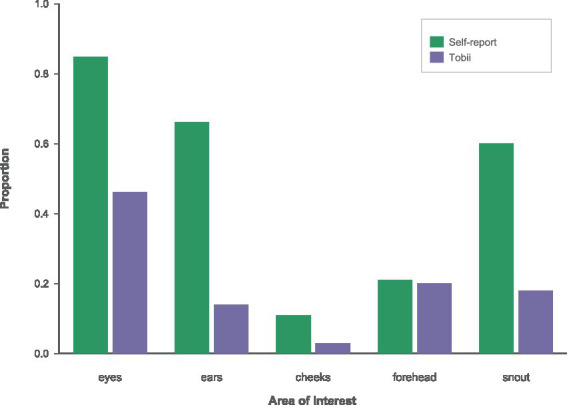
Self-report (green) vs. Tobii whole-fixation (purple) proportions combined for both breeds.

Self-reported attention and Tobii visit data showed both convergence and divergence across the five facial areas of interest (Figure 10). In self-reports (combined across breeds), participants most frequently indicated looking at the eyes (M = 0.85, 95% CI [0.81, 0.89]), followed by the ears (M = 0.66, 95% CI [0.61, 0.71]) and snout area (M = 0.60, 95% CI [0.54, 0.65]), with lower endorsement for the forehead (M = 0.21, 95% CI [0.17, 0.26]) and cheeks (M = 0.11, 95% CI [0.08, 0.15]). Tobii visit-share similarly prioritized the eyes (M = 0.46, 95% CI [0.44, 0.48]) but showed a markedly lower proportion of visits to the ears (M = 0.14, 95% CI [0.12, 0.15]) and a relatively greater allocation of gaze to the snout (M = 0.18, 95% CI [0.16, 0.19]) and forehead (M = 0.20, 95% CI [0.18, 0.21]), with minimal visits to the cheeks (M = 0.03, 95% CI [0.02, 0.04]). Thus, both measures converge in highlighting the eyes as the dominant focus of attention, but they diverge in that participants *report looking much more at the ears and somewhat less at the* snout and forehead than the objective eye-tracking data reflect, consistent with a dissociation between perceived and measured viewing behavior.

**Figure 11:**
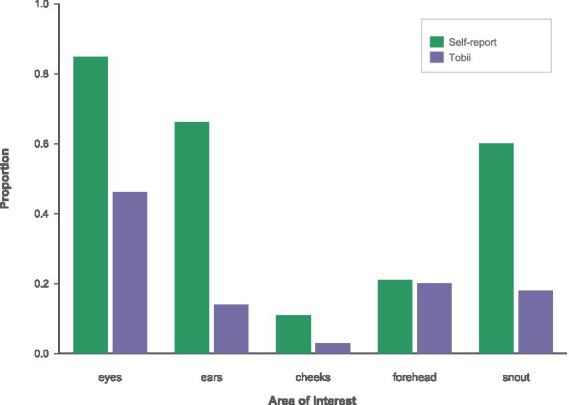
AOI proportions for normocephalic breed using self-report (green) vs. Tobii number of visits (purple) color scheme.

**Figure 12:**
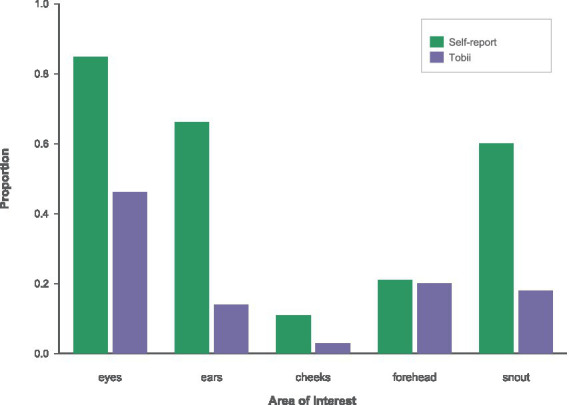
AOI proportions for brachycephalic breed using self-report (green) vs. Tobii number of visits (purple) color scheme.

This pattern is consistent across both normocephalic (Figure 11) and brachycephalic (Figure 12) panels, indicating that the basic viewing hierarchy and the disparity between perceived and measured attention generalize across different breeds.

#### 3.4.2 Self-reported proportions AOIs by breed

**Figure 13:**
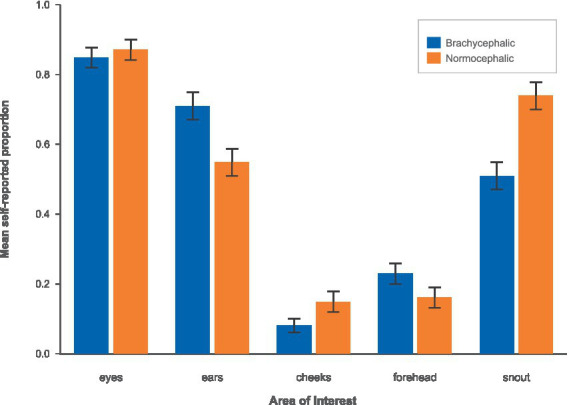
Self-report proportions by breed using the brachycephalic (blue) vs. the normocephalic (orange) color scheme.

We next summarized self-reported AOI mentions by breed (Figure 13). Self-reported attention to the dog’s eyes was high and did not differ between brachycephalic and normocephalic breeds (Brachycephalic: M = 0.85, 95% CI [0.79, 0.91]; Normocephalic: M = 0.86, 95% CI [0.79, 0.91]), t(42) = 0.11, *p* = 0.914, d = 0.03. Self-reported attention to the ears was higher for brachycephalic compared to normocephalic dogs (Brachycephalic: M = 0.73, 95% CI [0.65, 0.80]; Normocephalic: M = 0.60, 95% CI [0.51, 0.67]), t(42) = 2.36, *p* = 0.023, d = 0.71, with the 95% confidence interval for the difference [0.02, 0.25] indicating a medium-to-large effect.

For the cheeks, normocephalic dogs were descriptively rated as drawing more attention than brachycephalic dogs (Brachycephalic: M = 0.08, 95% CI [0.04, 0.13]; Normocephalic: M = 0.14, 95% CI [0.08, 0.20]), but the confidence interval for the difference included zero (95% CI [−0.13, 0.02]), and the effect was not statistically significant, t(42) = −1.55, *p* = 0.129, d = −0.47. Self-reported attention to the forehead was virtually identical across breeds (Brachycephalic: M = 0.22, 95% CI [0.14, 0.28]; Normocephalic: M = 0.20, 95% CI [0.15, 0.29]), t(42) = −0.19, *p* = 0.853, d = −0.06.

In contrast, participants reported looking more at the snout area of normocephalic dogs than at that of brachycephalic dogs (Brachycephalic: M = 0.52, 95% CI [0.44, 0.60]; Normocephalic: M = 0.68, 95% CI [0.60, 0.75]). The mean difference (Brachycephalic−Normocephalic) was −0.16, 95% CI [−0.27, −0.05], indicating a statistically significant and large effect, t(42) = −2.83, *p* = 0.007, d = −0.85.

#### 3.4.3 Tobii number of visits proportions by AOI and breed

**Figure 14:**
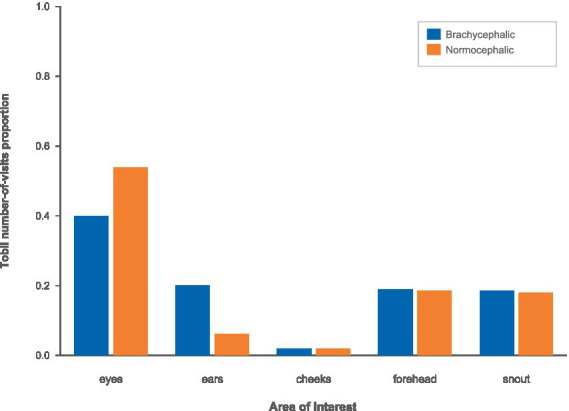
Tobii visit proportions by breed using the brachycephalic (blue) vs. the normocephalic (orange) color scheme.

Using Tobii’s number-of-visits, we computed each AOI’s visit share within participant-by-image and averaged these shares by breed and AOI (Figure 14). According to Tobii eye-tracking data, several notable breed differences emerged in how participants visually explored the dogs’ faces. The participants prioritized the eyes; they devoted a larger proportion of their Tobii-recorded visits to the eyes of normocephalic dogs (M = 0.50, 95% CI [0.47, 0.53]) than to the eyes of brachycephalic dogs (M = 0.42, 95% CI [0.39, 0.44]). The mean difference (brachycephalic−normocephalic) was −0.08 (95% CI [−0.12, −0.04]), indicating a large, statistically significant effect, t(42) = −3.87, *p* < 0.001, d = −1.17.

In contrast, participants showed the opposite pattern for the ears: a higher proportion of Tobii visits to the ears of brachycephalic dogs (M = 0.18, 95% CI [0.16, 0.20]) than to the ears of normocephalic dogs (M = 0.10, 95% CI [0.08, 0.12]). The mean difference was 0.08 (95% CI [0.05, 0.11]), which was statistically significant and large in magnitude, t(42) = 5.54, *p* < 0.001, d = 1.67.

For the cheeks, Tobii visit-share was very similar across breeds (brachycephalic: M = 0.03, 95% CI [0.02, 0.04]; normocephalic: M = 0.03, 95% CI [0.02, 0.04]). The mean difference was small and imprecisely estimated (Brachycephalic − Normocephalic = 0.00, 95% CI [−0.01, 0.02]), and the effect was not statistically significant, t(42) = 0.47, *p* = 0.640, d = 0.14.

Similarly, Tobii-based attention to the forehead did not differ meaningfully between breeds (brachycephalic: M = 0.20, 95% CI [0.18, 0.22]; normocephalic: M = 0.19, 95% CI [0.18, 0.22]). The mean difference was 0.00, 95% CI [−0.03, 0.04], t(42) = 0.27, *p* = 0.785, d = 0.08.

Finally, Tobii visit-share to the snout area was also highly similar for brachycephalic (M = 0.18, 95% CI [0.16, 0.19]) and normocephalic dogs (M = 0.17, 95% CI [0.16, 0.20]). The mean difference (−0.00, 95% CI [−0.03, 0.02]) was not statistically significant, t(42) = −0.35, *p* = 0.726, d = −0.11.

Taken together, the Tobii data indicate robust breed differences in the eyes and ears: participants looked relatively more at the eyes of normocephalic dogs and at the ears of brachycephalic dogs, whereas differences in the cheeks, forehead, and snout were negligible.

## 4 Discussion

The present study investigated how craniofacial morphology is associated with (i) overall attention to dog faces and (ii) prioritization of specific facial regions when humans interpret human-oriented facial signals. Participants provided self-reported AOI selections (i.e., which facial regions they believed they focused on) and, crucially, (iii) their visual attention was simultaneously recorded with an eye tracker for a comparison with their self-reported visual attention behavior.

### 4.1 Overall attention to dogs’ faces

The results strongly support our first hypothesis regarding processing intensity: participants allocated significantly more visual attention to images of brachycephalic dogs compared to normocephalic dogs across multiple converging measures. Brachycephalic dogs received 45–46% more visual attention than normocephalic dogs, nearly half again as much attention. This difference is not only statistically significant but also practically important for real-world interactions. For comparison, effect sizes of this magnitude are similar to the difference between glancing at a face for 2 s versus 4 s, or checking a feature 3 times versus 5 times during a brief encounter. Such pronounced differences in visual attention allocation likely have meaningful implications for how humans perceive and respond to these dogs in naturalistic settings, where rapid visual assessment often guides approach and interaction decisions (52).

One of the most notable aspects of our findings is the dual pattern of increased frequency and duration. Participants not only looked at brachycephalic faces more frequently, showing 46% more fixations and 42% more visits, but they also spent 45% more total time viewing these faces. This pattern of both more frequent and longer cumulative viewing suggests sustained and intensive visual processing rather than mere fleeting curiosity. This pattern contrasts with situations in which a high frequency of fixations is paired with short individual fixations, which usually indicate rapid scanning or difficulty in locating relevant information (53). In this case, the combination of more frequent revisits and longer cumulative viewing times suggests that participants were actively engaged in extracting and integrating information from brachycephalic faces. This interpretation is supported by research on face processing, which has shown that increased viewing time, along with more frequent eye movements, indicates effortful encoding when facial configurations deviate from typical structures (43). The brachycephalic craniofacial structure, characterized by its compressed proportions and altered feature spacing, may elicit this extended, iterative processing to interpret social signals that are more readily recognizable in typical canine faces.

The increased frequency of saccades in brachycephalic dogs (46% more) may reflect increased visual processing. Saccades are quick, automatic eye movements that shift the fovea to different locations, allowing for precise sampling of visual details (54). An increase in saccadic activity can indicate active information gathering and feature comparison as the visual system builds a coherent representation of the scene (55). In face processing research, higher saccade rates are associated with greater encoding effort, especially when faces display ambiguous or difficult-to-interpret signals (56). It is important to recognize that eye-tracking metrics such as saccade frequency are not direct equivalents of cognitive difficulty, but rather indicators of visual exploration patterns that may arise from multiple factors. The significant increase in saccades toward brachycephalic faces may indicate that participants actively compared and integrated information across multiple facial features, frequently moving their gaze between the ears, eyes, and snout area to decode expressions or intentions. Suggesting that a typical facial configurations may require more extensive visual sampling to extract relevant information. The automatic nature of saccadic movements makes them a sensitive indicator of cognitive processing demands. Participants likely were not consciously aware of making more eye movements, but their visual systems automatically increased saccade frequency to cope with the heightened processing challenge posed by brachycephalic facial morphology.

The absence of breed differences in average fixation duration, despite significant variation across various frequency-based measures, requires careful analysis. One possibility is that average fixation duration may not be the most effective metric for capturing processing differences in this context. Individual fixation durations are highly variable, as indicated by our large standard deviations. This variability can be influenced by moment-to-moment factors, such as the specific feature being examined, the amplitude of the preceding saccade, and unrelated factors like blinks or micro-adjustments (57). When these individual fixations are averaged, the resulting value may obscure systematic differences that could exist at finer temporal scales.

The disconnection between fixation frequency and duration, specifically, the significant increase in fixations on brachycephalic faces without a corresponding change in average fixation duration, reveals the visual sampling strategy being utilized. During face viewing, visual attention reflects a balance between exploration and exploitation (58). Exploration involves shifting gaze to gather information across different areas, as indicated by higher fixation counts. In contrast, exploitation involves longer fixations for deeper local processing, as evidenced by the extended gaze duration (59). The observed pattern suggests a shift toward exploration rather than exploitation, with participants distributing their attention more broadly across the features of brachycephalic faces through iterative sampling, rather than engaging in sustained analysis of specific regions. Instead of spending more time on individual features, which would increase the average duration, participants appear to adopt a strategy of briefly revisiting features for quick checks. This approach increases total viewing time through cumulative revisits rather than longer individual fixations.

This exploratory bias may arise from several factors. First, the compressed spatial arrangement and altered feature relationships in brachycephalic faces may disrupt configural processing mechanisms (60). As a result, it may be necessary to engage in more extensive spatial sampling to extract relational information that is typically processed holistically in normal facial configurations. This behavior aligns with research on complex visual scenes, where difficulties in integration, not in perceiving individual features, lead to more frequent revisits without necessarily extending the duration of individual fixations (61). The unique, compressed spatial arrangement of brachycephalic faces may pose integration challenges that are better addressed through repeated sampling rather than sustained observation. Second, uncertainty in interpreting emotional signals from unusual physical features may encourage participants to actively seek information. They tend to gather evidence across multiple characteristics rather than focusing deeply on a single cue (62). Third, distinctive traits, like proportionately large eyes and infant-like proportions, can lead to multiple competing areas of interest, promoting a more widespread approach to gathering information.

The exploration-exploitation framework highlights that an increased frequency of fixations does not necessarily mean that participants are experiencing processing difficulties. Instead, it suggests a different strategy for gathering information. The stable duration of fixations indicates that participants are not struggling to process local features; rather, they are efficiently sampling information from multiple locations. This exploratory mode seems to be an adaptive response to the unique morphological characteristics of brachycephalic facial structures. This could explain the increase in frequency while the average duration remains stable.

The analysis of AOIs revealed that the ears of brachycephalic dogs were the most prominent features, consistently demonstrating the largest effects across all frequency measures. The left ear exhibited the strongest effects, with Hedges’ g values ranging from 0.69 to 1.59 across the measures. This was followed by the right ear, left eye, forehead, and snout. Notably, the data on time to first visit indicated that the ears of brachycephalic dogs captured attention more quickly: the left ear attracted attention approximately 2.9 s earlier, and the right ear approximately 2.3 s earlier. In contrast, the right eye showed a delayed response in attention capture, taking about 1.7 s longer.

This temporal pattern, characterized by increased and sustained attention to the ears, indicates that these features are particularly noticeable or informative when processing brachycephalic faces. In typical dog faces, ear position and movement play important roles in communication (18). However, the altered proportions of brachycephalic skulls can make ear positions more ambiguous and harder to interpret, potentially requiring greater visual attention. Recent research has demonstrated that humans are sensitive to subtle changes in dog facial expressions, especially around the eyes and ears (1, 43). The compressed morphology of brachycephalic breeds can distort these essential signals. Additionally, the forehead and snout received significant attention, likely due to the marked morphological changes in brachycephalic breeds. The shortened muzzles and pronounced forehead bulges create visual configurations that deviate significantly from typical canine facial templates.

The right eye consistently displayed a different pattern, as normocephalic dogs received more attention across all frequency measures. Several factors may contribute to this asymmetry. The most parsimonious explanation is the subtle head tilt visible in the brachycephalic stimuli, which reduced the visibility of the left eye relative to the right (as discussed in the Limitations section). However, hemispheric lateralization may also play a role: in humans, face processing is predominantly a right-hemisphere function, with the left visual field (and thus the right side of a viewed face) typically receiving preferential attention (63). If participants processed the normocephalic faces using this standard lateralised strategy, while the altered geometry of brachycephalic faces disrupted the typical scanning pattern, this could account for the selective right-eye advantage observed only in normocephalic dogs. Regardless of the underlying mechanism, this asymmetry does not undermine the primary finding that brachycephalic faces garnered significantly more visual attention across most facial regions.

These findings support the idea that the extreme craniofacial structure of brachycephalic dog breeds, as demonstrated by morphometric studies (16, 19); and re-evaluated by Ref. (20), may make it more challenging for humans to understand and interpret their facial signals. This challenge may be associated with greater cognitive effort and more visual scanning. The compressed facial structure of these breeds alters the spatial arrangement of their facial features, potentially which may disrupt the visual processing strategies humans rely on to interpret canine expressions and intentions.

Human perception of dog faces is likely based on evolved or learned templates that recognize typical canine facial configurations. Specific arrangements of features can signal different emotional states or intentions (30). However, brachycephalic breeds differ significantly from these templates. Their shortened skulls compress the distance between features, while their prominent foreheads alter the facial triangle. Additionally, their reduced muzzles eliminate a key element of typical dogs’ expressiveness. These changes may make standard processing strategies ineffective, requiring observers to engage in more effortful and iterative visual sampling to form an interpretable representation of the dog’s face.

The observed results indicate that increased sampling without deeper processing of individual faces, along with heightened saccadic activity, suggests that observers are making greater efforts to integrate information from brachycephalic faces. However, they struggle to do this efficiently during single fixations. This interpretation aligns with broader research on atypical face processing, which shows that deviations from typical facial configurations lead to longer viewing times and more eye movements required for the recognition or judgment of expressions. In the context of human-dog interactions, the increased demand for processing facial cues may have significant welfare implications. Owners and veterinarians who find it challenging to interpret the facial signals of brachycephalic dogs may overlook important communications related to pain, distress, or anxiety (1, 8, 43). This concern is particularly salient given recent advances in the use of canine facial expressions as clinical indicators of pain (4); if the facial morphology of brachycephalic breeds renders such signals less legible to human observers, the clinical utility of expression-based pain assessment may be reduced in these breeds. This oversight could compromise both the quality of care for these dogs and the bond between humans and animals. Given the rising popularity of brachycephalic breeds despite their well-documented health issues, understanding how humans perceive and interpret these dogs’ facial signals is increasingly important for promoting responsible ownership and veterinary practices.

In summary, brachycephalic dogs received substantially more visual attention than normocephalic dogs, characterized by increased sampling frequency rather than deeper individual processing. This pattern has important implications for both the interpretation of specific facial features and human-dog communication more broadly.

### 4.2 Prioritization of specific facial AOIs

Our second hypothesis of feature prioritization, which posited that the eye area would be of major interest to human viewers, was not confirmed. While the eye region was predominantly examined in normocephalic dogs, the same could not be said for brachycephalic dogs. The eye tracking data revealed a distinct “central-feature” scanning strategy used by participants to decode canine facial cues.

Interestingly, while our previous study (5) revealed a high self-reported attention in the eyes and ears of both featured breeds, in the presented study, particularly the ears of the brachycephalic dogs attracted more attention than those of the normocephalic dogs. Interestingly, the gaze pattern suggested that participants inspected the eye and the central face of the Jack Russell Terriers, but in Boston Terriers, the gaze followed a clockwise track around the dog’s head. This could be explained by morphological differences: while Jack Russell Terriers display flopped ears, Boston Terriers typically have fully erect, bat-like ears (see the FCI standards for the Boston Terrier and Jack Russell Terrier). This could explain why, unlike other studies that reported a strong level of attention from human viewers to canine eyes (39, 43, 64), our findings revealed that this focus differs among cephalic types.

In other studies, dogs’ eyes were usually fixated on first, followed by the snout area (39).

These two AOIs were predominantly fixated upon (64). Our results indicated that, in both breeds, the forehead was the first area of interest to be inspected. It could be suggested that, since the forehead, with its high color contrast [proven to affect human viewers; see Ref. (65)], was always situated in the center of the photographs, the first viewing of this particular AOI in both breeds could be explained in this way. Alternatively, it can be theorized that the focus on the dogs’ foreheads was interpreted as a connection between the eyes and the ears, or as a search for the forehead crease. The more apparent forehead creases on the smooth-haired Boston Terriers, along with their steeper stop (the angle of the forehead to the caudal rostrum) and the higher contrast of the white markings to the darker fur (65) could have further drawn attention to the eye and forehead area of the brachycephalic dogs. The high number of saccades to the forehead and snout area regions in both breeds suggests that these areas draw the human gaze for inspection.

Despite fur type not being associated with a significantly altered ability to interpret affective cues of dogs (14), the rougher fur of the Jack Russell Terriers might have potentially obscured some features, resulting in increased attention in the eye area. This triangulation of gaze behavior suggests that while the snout area is visually prominent, the eye region serves as the primary anchor for information gathering. Still, more thorough research is needed to determine the exact effect that fur type has on the clarity of canine visual signals to humans.

### 4.3 Comparing self-estimation and objective attention patterns

The substantial discrepancies between participants’ self-reported attention and their objectively measured gaze behavior represent a critical methodological finding with important implications for understanding human-dog interaction. These discrepancies align with a growing body of research demonstrating that people have limited metacognitive awareness of their own visual attention patterns (66, 67).

While human focus on canine eyes has been documented in previous eye-tracking studies (42, 43), the present study reveals that objective attention to the eye region is even greater than participants consciously recall. This finding is consistent with research on metacognitive judgments, which shows that observers systematically underestimate their fixation frequency on highly salient features, particularly in face-processing tasks (67). The metacognitive literature suggests that people struggle to accurately report where they looked because eye movements occur largely automatically and below conscious awareness (66). When forced to retrospectively report their attention allocation, participants tend to rely on cognitive heuristics about what “should” be important rather than accurate memories of their actual gaze behavior.

Similarly, the informative value of canine ears appears substantially higher than people anticipate based on their self-reports. This is particularly notable given that previous eye-tracking studies on canine faces (42, 71) have not reported extensive inspection of the ear region, suggesting that our finding of elevated ear attention may be specific to breed-related morphological differences. Recent work on visual attention and self-awareness has demonstrated that people systematically overestimate their attention to features they consciously recognize as “important” while underestimating attention to features processed more automatically or peripherally (68). Our previous research (14) demonstrated that ear shape influences humans’ ability to distinguish affective states from canine faces, suggesting that ear configuration carries diagnostic information even when observers are not consciously aware of attending to this region.

The most striking discrepancy emerged for the snout area, where recorded visual attention was substantially lower than participants’ self-reports, despite two stimulus images showing dogs with open mouths and visible tongues, which were in contrast with the surrounding areas. This finding contradicts previous studies reporting strong fixations on nasal and oral regions (39, 43, 71). This pattern is consistent with research on food visual attention, showing that people overestimate their focus on features they consider culturally or contextually salient while actual gaze patterns reveal different priorities (68). The overestimation may reflect several cognitive biases documented in the metacognitive literature. First, the distinctiveness heuristic suggests that unique or unusual features (such as visible tongues) are more memorable and thus more likely to be reported even when they were not extensively fixated (66). Second, the social significance of the mouth region in human communication may create a cognitive bias, leading participants to believe they attended to this region more than they actually did.

One plausible explanation is that participants scanned regions adjacent to the mouth (which were registered as fixations on the cheeks in our AOI coding) while still processing mouth information via peripheral vision. This interpretation aligns with research demonstrating that peripheral vision contributes substantially to face processing beyond focal attention (67), and that observers often extract critical facial information from areas they do not directly fixate. However, this explanation remains speculative and highlights the need for future research with more granular AOI definitions that distinguish between the nose, mouth, and perioral regions.

The discrepancy between self-reported and objective attention also manifests in overestimation of attention to socially salient features. This phenomenon has been documented in human face processing research (34) and extends to cross-species face perception in our study. Rogers et al. (34) demonstrated that humans overestimate their focus on distinct facial regions, such as conspecifics’ eyes, by alternating attention between the region and scanning it rather than sustaining focus as they subjectively perceive. Our findings suggest that this metacognitive bias generalizes to dog faces, particularly in regions considered socially or emotionally communicative (e.g., eyes, mouth).

Several converging factors may explain why participants’ self-reports systematically underestimated the breadth of regions they actually fixated. First, the majority of saccadic eye movements and fixation shifts occur automatically and below conscious awareness (57, 66), which fundamentally limits observers’ ability to accurately reconstruct their own scanning behavior after stimulus offset. When forced to report retrospectively, participants likely relied on top-down heuristics about which facial features “should” be informative, such as the mouth or snout, which carry high social significance in human communication rather than drawing on veridical memories of their gaze trajectories. This heuristic reliance may be compounded by a distinctiveness bias: visually striking features, such as a visible tongue or open mouth, tend to be encoded more memorably and are therefore over-represented in retrospective reports even when they were not extensively fixated (67). At the same time, participants may have extracted meaningful information from regions processed via peripheral rather than foveal vision, leading them to report attending to areas they never directly fixated but nonetheless perceived (61). Taken together, these factors point to a fundamental constraint on metacognitive access to one’s own visual scanning behavior: people systematically misjudge not only how long they looked at specific regions, but also which regions they looked at (34, 67). These findings reinforce the need for objective gaze measurement in studies of cross-species face perception, as self-report alone may produce a misleading picture of how humans actually allocate visual attention when interpreting canine facial signals.

The convergence of evidence from metacognitive research and eye-tracking studies reveals a fundamental challenge in visual attention research: simply fixating on a particular area does not necessarily mean that it is important for decision-making. Participants may focus on visually striking areas that provide little information, or they may extract crucial details from brief glances at the periphery (66). This distinction between overt attention (where we look) and covert attention (the information we gather) is well documented in the metacognitive literature but often overlooked in applied animal behavior research.

Combining objective eye-tracking metrics with subjective self-reported assessments of which areas participants find most informative is a crucial step in validation (68). This methodological triangulation allows us to differentiate between mere visual attention (overt fixations) and perceived diagnostic value (subjective importance). It strengthens the interpretability of our findings and provides convergent evidence about which facial features truly contribute to accurate emotion recognition in both brachycephalic and normocephalic dog breeds. The discrepancies uncovered in this study highlight the importance of using multi-method approaches in animal behavior research and suggest that relying solely on self-report measures can lead to misleading conclusions about how humans actually process canine facial signals.

Future research should investigate the mechanisms behind metacognitive failures in cross-species communication. Specifically, studies that utilize real-time verbal protocols or confidence ratings during observation, rather than relying on retrospective self-reports, might clarify whether metacognitive errors occur during the encoding, consolidation, or retrieval of attention-related information. Furthermore, comparing expert populations, such as veterinarians and dog trainers, with novice observers could help determine if experience enhances the accuracy of reporting visual attention to canine faces.

### 4.4 Limitations

While this study provides objective evidence regarding the visual processing of canine facial morphology, several limitations should be considered when interpreting the results.

First, methodological design choices should be acknowledged. To ensure replicability with Eretová et al. (5), images were presented in a fixed order without counterbalancing, which does not control for potential order or fatigue effects. Additionally, the sequential image-questionnaire format, while necessary for collecting self-reported validation data, may have encouraged strategic rather than spontaneous viewing patterns. Since all participants experienced identical procedures, these factors should affect both breeds equally; however, future research should use counterbalanced presentation orders and explore differences between task-directed and spontaneous viewing conditions.

Second, the stimuli used in this study were static photographs cropped to focus exclusively on the dogs’ faces. While this controlled body language and background distractions, it lacks the temporal dynamics inherent in real-world canine communication. Facial expressions in dogs are often fleeting and integrated with whole-body signals; therefore, the gaze patterns observed here may differ from those elicited during dynamic interactions or video stimuli, where movement likely captures attention differently. The materials also pose limitations due to the uneven angles of the canine faces toward the camera, making a precise interpretation of right–left bias somewhat difficult. Moreover, the ears of our chosen brachycephalic breed, the Boston Terrier, are unusually large and noticeably erect; thus, participants’ strong interest in them may reflect a bias stemming from the breed’s ear conformation. Additionally, two of the photographs, both featuring Boston Terriers, showed a green wall in the background, while the rest of the dataset (the remaining two Boston Terrier photographs and all four Jack Russell Terrier photographs) showed a white tiled floor. Both backgrounds were solid, with little to no disturbances, and the eye-tracking technology examined gaze patterns only within the specific AOIs, reducing the potential effects of the differing backgrounds; however, some effects on the participants cannot be ruled out.

Third, the study compared only two specific breeds to represent the extremes of craniofacial morphology: the Boston Terrier (brachycephalic) and the Jack Russell Terrier (normocephalic). Consequently, the findings may not be fully generalizable to all brachycephalic or normocephalic breeds, as individual breed features (e.g., skin folds, pigmentation, or ear shape) could influence gaze allocation distinct from skull shape alone.

Fourth, the participant sample consisted of undergraduate students from a technological college with a mean age of approximately 23 years. This demographic homogeneity limits the generalizability of the findings to the broader population. Notably, data from one significantly older participant had to be excluded from the analysis, preventing us from exploring potential age-related differences in how canine signals are processed. In addition, the sample was predominantly male (63.6%). Although our exploratory analyses revealed no significant gender effects on eye-tracking measures, the unbalanced composition may have limited statistical power to detect subtle gender-related differences in visual attention to canine faces. Prior research has reported that women tend to score higher on measures of empathy toward animals (69) and may attend more closely to emotionally expressive facial features, suggesting that a more gender-balanced sample could yield additional nuance regarding how observer characteristics interact with breed morphology. Future studies should aim for balanced samples across gender and a broader age range to strengthen generalizability. Finally, technical constraints resulted in the exclusion of nearly one-third of the collected eye-tracking data due to tracking errors, blinks, or fixation durations below the threshold. While this rigorous data cleaning ensures the validity of the reported fixations, it suggests that future protocols might benefit from head-mounted eye trackers or more robust tracking environments to capture a more complete dataset. The 60 Hz sampling rate of the Tobii Pro Spark is appropriate for fixation-focused analyses but may underestimate rapid saccadic movements compared to higher-frequency systems. However, this limitation applies uniformly across conditions and does not compromise our comparative analyses between breeds.

## 5 Conclusion and future work

This study is the first to use eye-tracking technology to objectively measure how craniofacial morphology affects human visual attention during the interpretation of canine signals. Our findings reveal that brachycephalic dog faces, such as those of Boston Terriers, were associated with greater cognitive and visual demands than those of normocephalic breeds. Viewers adopted an ‘intensive sampling’ strategy characterized by increased fixation frequencies and longer cumulative viewing times, indicating that extreme brachycephaly may obscure critical facial cues for interspecies communication.

Additionally, the research underscores the importance of objective gaze measurement over self-reporting. Substantial discrepancies emerged between self-reported and objectively measured attention: participants fixated more on the eye region than they believed, while overestimating their attention to the snout area. These results highlight the communicative challenges posed by extreme breeding and indicate that humans face greater difficulty in interpreting the faces of short-nosed dogs.

Future research should examine a wider range of breeds spanning the cephalic spectrum to assess whether the “intensive sampling” strategy observed here represents a general response to brachycephaly. In addition, studies should recruit more diverse participant groups, such as children, older adults, and canine professionals, to examine whether age or experience influences attention to specific facial regions. Finally, future protocols may benefit from the use of head-mounted eye trackers or more controlled tracking environments to ensure more complete and reliable data capture.

## Data Availability

The data analyzed in this study is subject to the following licenses/restrictions: the data will be disclosed upon reasonable request. Requests to access these datasets should be directed to annazam@gmail.com.
